# Novel Mutations and Genes That Impact on Growth in Short Stature of Undefined Aetiology: The EPIGROW Study

**DOI:** 10.1210/jendso/bvaa105

**Published:** 2020-09-10

**Authors:** Reena Perchard, Philip George Murray, Antony Payton, Georgina Lee Highton, Andrew Whatmore, Peter Ellis Clayton

**Affiliations:** 1 Developmental Biology & Medicine, Faculty of Biology, Medicine & Health, University of Manchester and Manchester Academic Health Science Centre, Manchester, United Kingdom; 2 Department of Paediatric Endocrinology, Manchester University NHS Foundation Trust, Manchester, United Kingdom; 3 Informatics, Imaging & Data Science, Faculty of Biology, Medicine & Health, University of Manchester, Manchester, United Kingdom

**Keywords:** growth, short stature, short stature of undefined aetiology, idiopathic short stature, short stature syndrome, short stature mutations

## Abstract

**Background:**

Children with short stature of undefined aetiology (SS-UA) may have undiagnosed genetic conditions.

**Purpose:**

To identify mutations causing short stature (SS) and genes related to SS, using candidate gene sequence data from the European EPIGROW study.

**Methods:**

First, we selected exonic single nucleotide polymorphisms (SNPs), in cases and not controls, with minor allele frequency (MAF) < 2%, whose carriage fitted the mode of inheritance. Known mutations were identified using Ensembl and gene-specific databases. Variants were classified as pathogenic, likely pathogenic, or variant of uncertain significance using criteria from the American College of Medical Genetics and Genomics and the Association for Molecular Pathology. If predicted by ≥ 5/10 algorithms (eg, Polyphen2) to be deleterious, this was considered supporting evidence of pathogenicity. Second, gene-based burden testing determined the difference in SNP frequencies between cases and controls across all and then rare SNPs. For genotype/phenotype relationships, we used PLINK, based on haplotype, MAF > 2%, genotype present in > 75%, and Hardy Weinberg equilibrium *P* > 10^–4^.

**Results:**

First, a diagnostic yield of 10% (27/263) was generated by 2 pathogenic (nonsense in *ACAN*) and a further 25 likely pathogenic mutations, including previously known missense mutations in *FANCB*, *IGFIR*, *MMP13*, *NPR2*, *OBSL1*, and *PTPN11*. Second, genes related to SS: all methods identified *PEX2*. Another 7 genes (*BUB1B, FANCM, CUL7, FANCA, PTCH1, TEAD3, BCAS3*) were identified by both gene-based approaches and 6 (*A2M, EFEMP1, PRKCH, SOS2, RNF135, ZBTB38*) were identified by gene-based testing for all SNPs and PLINK.

**Conclusions:**

Such panels improve diagnosis in SS-UA, extending known disease phenotypes. Fourteen genes related to SS included some known to cause growth disorders as well as novel targets.

Height is one of the most heritable human traits [1–3]. Adult height is a classic polygenic trait, determined by numerous deoxyribonucleic acid (DNA) sequence variants at many genetic loci. Genome-wide association studies (GWAS) have identified hundreds of genetic variants that influence adult height [4], with the NHGRI-EBI Catalog [5] listing 679 variants with *P* < 5 × 10^-8^ in 20 studies. In addition, age at onset of puberty and age at maximal growth spurt and skeletal maturation in childhood are also largely under genetic control [6–9]. Therefore, it is highly likely that there is an underlying genetic cause (which may be monogenic or polygenic) in many children with short stature of undefined aetiology (SS-UA), often termed *idiopathic short stature* (ISS) in the clinical setting.

EPIGROW [10] was a cross-sectional prospective epidemiological study conducted at 64 sites in 9 European countries to identify genes from a candidate list that may be associated with short stature (SS). It included prepubertal children with SS with no identified cause and normal growth hormone levels following stimulation testing. Next-generation deep sequencing (NGS) was performed in 263 individuals with SS and in 263 ethnically matched controls, on exons, exon–intron junctions, and promoter regions of 232 candidate genes. A case-control approach was used to identify genetic associations of SS by comparing the frequencies of single nucleotide polymorphisms (SNPs) and insertion-deletions (indels) between cases and controls.

This identified 2 SNPs in zinc-finger and BTB domain-containing protein 38 (*ZBTB38)* with a significantly lower frequency in cases than controls. Two indels, 1 in nuclear factor kappa B1 (*NFKB1*) and 1 in insulin-like growth factor-1 (IGF-1), were also associated with SS. *ZBTB38* and *NFKB1* are both involved in the regulation of transcription, implying that other pathways not directly related to the growth hormone (GH)–IGF-1 axis have an impact on SS.

These original findings resulted from single-marker analysis of SNP/indel frequency between cases and controls. We have now analyzed the data using new approaches. In this study, our first aim was to identify mutations in genes known to be related to SS conditions, thereby establishing diagnoses. Our second aim was to determine genes that displayed differences in overall SNP frequency, using gene-based burden testing approaches. Thirdly, we aimed to define associations between SNPs and particular growth characteristics/biochemical measures of IGF status. By identifying genes that featured in 1 or more of these analyses, we aimed to determine genes that are of particular interest in SS-UA.

## Methods

### Patient Population

A total of 263 patients were included in the genetic studies of EPIGROW and were therefore analyzed in this study. Cases within the EPIGROW study were paired with ethnically matched anonymized controls using a set of 95 autosomal SNPs that cluster samples into ethnic groups (Berkeley HeartLab, Inc, San Francisco, CA).

Details of the patient population have been published previously [10]. The study was conducted in compliance with independent ethics committee informed consent regulations, the Declaration of Helsinki, and International Conference on Harmonization and Good Epidemiological Practice Guidelines. The study adhered to all local regulatory requirements, including those related to genetic analyses. Signed informed consent forms (including consent for genetic research) were obtained from parents or legally acceptable representatives (and the child when applicable) before participation in the study. All patients were prepubertal, aged > 2 years, had at least 1 normal peak response to a GH stimulation test (peak GH > 7 μg/L) over the previous 12 months, a height standard deviation score (SDS) ≤ -2.5 according to local growth charts, and no identified cause of SS. Data collected included age, sex, birth length, birthweight, and most recent height SDS. Height data were standardized according to British 1990 growth reference data [11].

Children were excluded if there was a known cause for their SS, for example, skeletal dysplasia [10]. Biological analysis in the original EPIGROW cohort had revealed that the prevalence of IGF-1 deficiency was high at 53%. All these cases were included in the current study.

### Data Collection

Alongside medical history and clinical examination, demographic and auxological data were collected at an initial visit. Blood samples were obtained for DNA extraction and for serum IGF-1, IGF-binding protein-3 (IGFBP-3), and acid-labile subunit (ALS) measurements in standard assays [10].

### Genetics

Next-generation deep resequencing was performed on 1.35 Mb of genomic DNA on 232 candidate genes, selected on the basis of those that encoded proteins known to function in the GH–IGF signal transduction axis, to contain variants associated with height variation in the general population, or to link to known SS conditions [10] (69 genes, listed in Supplemental Table 1). Genetic data have been reported using Gene build NCBI 38 (October 2018). All Supplementary Material and Figures are located on an online research repository, available at [12].

### Detecting Mutations

To identify potentially pathogenic variants, we selected those which were present in cases but not controls, were exonic, had a MAF < 2%, and where carriage of the variant allele fitted the mode of inheritance of the known SS disorder.

To predict whether a variant had a deleterious effect on the protein, we used 10 *in silico* algorithms. These were Polyphen2, SIFT, FATHMM, LRT, M-CAP, MetaLR, MetaSVM, Mutation Assessor, Mutation Taster, and Provean. If a variant was predicted by ≥ 5 algorithms to be deleterious, the prediction was considered as supporting evidence of pathogenicity.

To identify known mutations, a combination of Ensembl and Leiden Open Variation Database (to access a range of gene-specific databases, eg, ClinVar) was used.

To classify variants, criteria recommended by the American College of Medical Genetics and Genomics and the Association for Molecular Pathology were applied [13]. This allowed for the classification of potentially pathogenic variants into the following categories: pathogenic, likely pathogenic, benign, and likely benign. Where contradictory evidence was obtained (ie, where the variant met the other criteria to be classed as likely pathogenic but was predicted deleterious by < 5 algorithms), the classification of variants of uncertain significance (VUS) was used.

### Gene-based Burden Testing

Whilst the original EPIGROW study [10] had compared frequencies of individual SNPs/indels between cases and controls, we determined the enrichment of particular genes for SNPs/indels between cases and controls. We achieved this by comparing the total frequencies of SNPs/indels in each of the 232 candidate genes. Additionally, we aimed to determine the enrichment of particular genes for only rare SNPs/indels.

### Single Nucleotide Polymorphism Carriage Frequencies for All Variants

Sequence data from EPIGROW were used to determine the difference in frequencies of SNPs/indels in each of the 232 genes between patients and controls. Single nucleotide polymorphism/indel frequency was assessed for carriage of homozygous and heterozygous variants compared to wild type, with significance evaluated in the chi-squared test (see Supplemental Table [12]). The resultant *P*-values were adjusted using a Bonferroni correction, and a corrected *P*-value of < 0.05 was considered significant.

### Single Nucleotide Polymorphism Carriage Frequencies for Rare Variants

Qualifying variants were determined by applying a filter to include only those SNPs with an MAF < 2%. The above analysis to assess SNP carriage frequency was then repeated.

### Single Nucleotide Polymorphisms Associated with Phenotypic Characteristics

Prior to defining associations between individual SNPs and growth characteristics/measures of IGF status, a number of quality control measures were applied. Genotype call rates ≥ 75% for individuals and SNPs were used for downstream quality control and analysis. To determine ethnicity, data were linkage disequilibrium (LD) pruned using PLINK software (Version 1.9) [14] and regions of high LD (such as human leukocyte antigen) were removed. Data were converted to EIGENSTRAT format [15] and ethnicity was inferred from autosomal SNPs using the SNPWeights (version 2.1) program [16] and the SNPWeight “snpwt.CO” reference panel (built from YRI, CEU, CHB, and CHD samples from HapMap3). Individuals with homogeneous (≥ 80%) Caucasian ancestries were selected (246 subjects) and SNPs with Hardy-Weinberg Equilibrium *P* ≤ 10^–4^ (603 SNPS) or outlying heterozygosity ≥ 3 standard deviations from the mean were removed.

Linear regression analyses were performed using PLINK [17]. The growth characteristics analyzed were standing height, sitting height, birthweight, birth length, target height (based on parental heights), and head circumference, as well as IGF-1, IGFBP3, and ALS levels. Single nucleotide polymorphisms with MAF > 2% were analyzed.

After adjusting for LD, there were 1650 variants with a frequency > 2%. Based on this, and a standard significance level of 0.05, a *P*-value < 3.03 × 10^–5^ was considered significant (0.05/1650). When assessing for overlaps between the results of this analysis and both gene-based burden testing approaches, a less stringent threshold was used. An unadjusted *P*-value of ≤ 0.001 was considered significant.

## Results

### Participants

The baseline characteristics of the participants have been previously described and are shown in Table 1. Participants were predominantly male (61%), had a mean age of 8.4 years, and were significantly short with a mean height of -2.9 SDS. Further auxological characteristics and the distributions of IGF-1, IGFBP-3, and ALS concentrations have been previously described [10].

**Table 1.** Baseline characteristics of participants with SS SDS

Characteristics	n	Mean (SD)	95% CI
Age (y)	259^*a*^	8.4 (3.2)	8.0–8.8
Male (%)	61		
Preterm (<37 weeks)	41		
Birth length SDS	214	−0.7 (1.3)	−0.9 to −0.5
SGA for weight (birthweight <2 SDS)	26		
Birth weight SDS	253	−0.7 (1.2)	−0.8 to −0.5
SGA for length (birth-length <-2 SDS)	27		
Microcephalic (visit 1 head circumference < -2 SDS)	110		
≥1 dysmorphic sign	38		
≥1 parent with height SDS <2	96		
Weight SDS	258	−2.5 (1.2)	−2.7 to −2.4
Bone age (y)	250	6.1 (3.0)	5.7–6.5
Height velocity (cm/y)	199	5.0 (1.5)	4.7–5.2
Sitting height SDS	165	−2.2 (2.4)	−2.6 to −1.8
Standing height SDS	255	−2.9 (0.7)	−3.0 to −2.8
Midparental height SDS	248	−1.3 (0.8)	−1.4 to −1.2

The characteristics of participants with SS are shown in this table; the participants were predominantly male, were significantly short with a mean height SDS of -2.9 and had a mean IGF-1 of -2 SDS.

Abbreviations: SDS, standard deviation score; SGA, small for gestational age.

^*a*^263 participants were included in the genetic analysis and 259 in the biological analysis.

### Mutations Identified

We identified 27 variants of interest (Table 2) in 27 patients, giving a potential diagnostic yield of up to 10% (27/263).

**Table 2.** Pathogenic and likely pathogenic variants

Gene	SNP	Amino Acid Change	RS Number (Where Available)	Nonsense/Missense	Classification	Variant Status
ACAN	c.2023C > T(ENST_00000439576)	ENSP00000271628.8:p.Gly328AlafsTer52		Nonsense	Pathogenic	Heterozygous
ACAN	c.2149G > T(ENST_00000439576)	ENSP00000263726.2:p.Cys92Tyr		Nonsense	Pathogenic	Heterozygous
FANCB	c.2452A > G(ENST_00000398334)	ENSP00000263726.2:p.Arg235Gln	rs200161949	Missense	Likely pathogenic	Homozygous
GDF5	c.1465C > A(ENST_00000374372)	ENSP00000328181.4:p.Asp75Ala		Missense	Likely pathogenic	Heterozygous
GH1	c.75T > G(ENST_00000323322)	ENSP00000312673.5:p.Ser25Arg		Missense	Likely pathogenic	Heterozygous
GNAS	c. 2150 A > G(ENST_00000371100)	-		Missense	Likely pathogenic	Heterozygous
GNAS	c.1581C > A(ENST_00000371100)	-		Missense	Likely pathogenic	Heterozygous
HRAS	c.481C > A(ENST_00000451590)	ENSP00000363489.3:p.Gln489Lys		Missense	Likely pathogenic	Heterozygous
IGF1R	c.4009C > T(ENST_00000268035)	ENSP00000354720.4:p.Glu183GlyfsTer11	rs141802822	Missense	Likely pathogenic	Heterozygous
IGF1R	c.3595G > A(ENST_00000268035)	-	rs886044448	Missense	Likely pathogenic	Heterozygous
IGF1R	c.3433G > A(ENST_00000650285)	ENSP00000302237.3:p.Ala174ProfsTer57	rs769418210	Missense	Likely pathogenic	Heterozygous
IGF1R	c.1162G > A(ENST_00000650285)	ENSP00000302237.3:p.Ser284AlafsTer8		Missense	Likely pathogenic	Heterozygous
IGF1R	c.4066G > A(ENST_00000650285)	ENSP00000302237.3:p.Ser463%3D		Missense	Likely pathogenic	Heterozygous
IKBKG	c.161G > A(ENST_00000594239)	ENSP00000264731.3:p.Thr566Met	rs782813189	Missense	Likely pathogenic	Homozygous
LHX4	c.704G > A(ENST_00000263726)	ENSP00000352514.5:p.Gly447Arg		Missense	Likely pathogenic	Heterozygous
LHX4	c1121T > C(ENST_00000263726)	ENSP00000341083.2:p.Phe321%3D		Missense	Likely pathogenic	Heterozygous
LHX4	c.275G > A(ENST_00000263726)	-		Missense	Likely pathogenic	Homozygous
MMP13	c.1372C > G(ENST_00000260302)	ENSP00000260302.3:p.Tyr405Cys	-	Missense	Likely pathogenic	Heterozygous
MMP13	c.1214A > G(ENST_00000260302)	-	rs190896822	Missense	Likely pathogenic	Heterozygous
NOG	c.224A > C(ENST_00000332822)	ENSP00000326819.3:p.Arg818Gly	-	Missense	Likely pathogenic	Heterozygous
NPR2	c.2723T > C(ENST_00000342694)	ENSP00000260302.3:p.Asp258%3D	rs369313283	Missense	Likely pathogenic	Heterozygous
NPR2	c.2720C > T(ENST_00000342694)	ENSP00000260302.3:p.Lys170Thr	rs1311857509	Missense	Likely pathogenic	Heterozygous
NPR2	c.2678T > C(ENST_00000342694)	ENSP00000323421.3:p.Leu1155ArgfsTer57	-	Missense	Likely pathogenic	Heterozygous
OBSL1	c.4005C > A(ENST_00000404537)	ENSP00000340944.2:p.Gly60Ala	rs375716830	Missense	Likely pathogenic	Homozygous
PTPN11	c.179G > C(ENST_00000351677)	ENSP00000341615.7:p.Arg675Ter	rs397507509	Missense	Likely pathogenic	Heterozygous
RUNX2	c.1381G > C(ENST_00000371438)		rs765089321	Missense	Likely pathogenic	Heterozygous
TP63	c.1697C > T(ENST_00000264731)		rs745687224	Missense	Likely pathogenic	Heterozygous

Variants classified as pathogenic and likely pathogenic according to American College of Medical Genetics and Genomics guidelines criteria and Association for Molecular Pathology guidelines criteria are shown.

The phenotype characteristics of these patients are illustrated in Table 3. Two of these were classified as pathogenic; these were previously known nonsense mutations in *ACAN*. An additional 25 were classified as likely pathogenic; these were missense mutations in *FANCB*, *GDF5*, *GH1*, *GNAS*, *HRAS*, *IGF1R*, *IKBKG*, *LHX4*, *MMP13*, *NOG*, *NPR2*, *OBSL1*, *PTPN11*, *RUNX2*, and *TP63*.

**Table 3.** Phenotype characteristics

SNP	Sex	Age at Visit 1	Dysmorphism	Heterozygous/ Vomozygous	Gene	Height SDS	Sitting Height SDS	Target Height SDS	BMI SDS	HC SDS	Birthweight SDS	Birth length SDS	Mean IGF-1	Mean IGFBP3	ALS
													SDS	SDS	
c.2023C > T(ENST_00000439576)	Female	10.67	Short neck	Heterozygous	ACAN	-4.31	–	-2.29	0.33	–	–	–	-0.13	-0.97	15.0 (4.2–13.0)
c.2149G > T(ENST_00000439576)	Male	6.83	High palate	Heterozygous	ACAN	-4.02	-3.18	-2.15	-1.02	-0.95	-2.25	–	-2.41	-1.07	11.0 (2.3–11.0)
c.2452A > G(ENST_00000398334)	Male	9.67	Nil	X-linked, hemizygous	FANCB	-2.15	–	-1.2	0.73	-1.58	-0.33	-1.61	-1.57	-1.61	7.3 (2.3–11.0)
c.1465C > A(ENST_00000374372)	Male	13	Nil	Heterozygous	GDF5	-2.53	-3.22	-1.08	-0.55	-0.96	0.86	-0.02	-1.57	-2.18	11.0 (5.6–16.0)
c.75T > G(ENST_00000323322)	Male	7.83	Brachydactyly	Heterozygous	GH1	-2.85	–	-1.72	-0.51	–	-1.06	-1.61	-1.34	-1.32	13.0 (2.3–11.0)
c. 2150 A > G(ENST_00000371100)	Male	5.75	Nil	Heterozygous	GNAS	-2.99	–	-1.86	-0.02	–	-1.08	-1.7	-3.94	-2.13	7.9 (2.3–11.0)
c.1581C > A(ENST_00000371100)	Male	7.42	Nil	Heterozygous	GNAS	-1.81	-1.2	-0.29	-0.56	-0.75	0.9	0.48	-1.8	-1.89	–
c.481C > A(ENST_00000451590)	Female	9.92	Nil	Heterozygous	HRAS	-2.24	–	-2.46	0.4	–	-1.24	-1.72	-1.56	-1.25	12.0 (4.2–13.0)
c.4009C > T(ENST_00000268035)	Male	3.58	Nil	Heterozygous	IGF1R	-2.09	-1.9	-1.51	-0.52	-3.42	-0.41	-0.17	-1.65	0.11	10.0 (1.9–10.0)
c.3595G > A(ENST_00000268035)	Male	14.5	Nil	Heterozygous	IGF1R	-4.23	-4.66	-2.23	-0.73	-3.03	-1.44	-0.84	-0.37	-1.16	18.0 (5.6–16.0)
c.3433G > A(ENST_00000650285)	Male	10.83	Nil	Heterozygous	IGF1R	-3.06	-2.92	–	-0.9	–	–	–	-2.11	-2.08	9.4 (4.2–13.0)
c.1162G > A(ENST_00000650285)	Male	14.25	Nil	Heterozygous	IGF1R	-3	-3.56	-0.84	-0.45	-0.57	0.5	-0.02	-3.73	-3.02	11.0 (5.6–16.0)
c.4066G > A(ENST_00000650285)	Male	9.17	Nil	Heterozygous	IGF1R	-3.18	–	-2.51	0.51	–	-0.53	–	-2.57	-1.25	12.0 (4.2–13.0)
c.161G > A(ENST_00000594239)	Male	7.83	Nil	Homozygous	IKBKG	-3.07	-4.38	-0.55	-0.64	-2.43	0.04	0.72	-2.63	-2.29	7.8 (2.3–11.0)
c.704G > A(ENST_00000263726)	Male	9.92	Nil	Heterozygous	LHX4	-2.92	–	–	-0.94	-1.72	-0.64	-1.61	-3.13	-1.53	9.5 (4.2–13.0)
c1121T > C(ENST_00000263726)	Male	7	Nil	Heterozygous	LHX4	-2.56	–	-1.94	-2.02		-0.32	-1.02	-2.46	-1.97	8.8 (2.3–11.0)
c.275G > A(ENST_00000263726)	Male	4.33	Nil	Homozygous	LHX4	-3.95	-3.99	-1.37	-0.14	-2.3	-0.8	-0.52	-2.42	-2.46	8.1 (1.9–10.0)
c.1372C > G(ENST_00000260302)	Male	9.08	Nil	Heterozygous	MMP13	-2.78	-2.37	-1.28	-3.39	-3.53	-1.33	-1.52	-2.82	-2.36	8.6 (4.2–13.0)
c.1214A > G(ENST_00000260302)	Male	7	Nil	Heterozygous	MMP13	-2.7	–	-2.52	-1.15	-4.2	-0.28	–	0.41	-0.41	–
c.224A > C(ENST_00000332822)	Male	4.92	Nil	Heterozygous	NOG	-3.01	-3.12	-1.46	-0.87	-1.8	-0.8	-0.76	-1.86	-2.12	4.9 (1.9–10.0)
c.2723T > C(ENST_00000342694)	Female	5.75	Large forehead, depressed nasal bridge	Heterozygous	NPR2	-4.12	–	-2.4	0.06	-3.22	0.21	–	-4.44	-3.69	3.2 (2.3–11.0)
c.2720C > T(ENST_00000342694)	Male	8.75	Nil	Heterozygous	NPR2	-2.88	-3.28	-1.86	1.05	-0.06	0.22	1.18	-2.97	-1.88	7.3 (4.2–13.0)
c.2678T > C(ENST_00000342694)	Male	5.33	Low-set ears	Heterozygous	NPR2	-2.15	–	-1.2	0.73	-1.58	-0.33	-1.61	-1.57	-1.61	7.3 (2.3–11.0)
c.4005C > A(ENST_00000404537)	Female	10.08	Nil	Homozygous	OBSL1	-2.47	–	0.11	0.04	-0.61	0.44	0.21	-3.08	-1.94	12.0 (4.2–13.0)
c.179G > C(ENST_00000351677)	Male	7.75	R testis not palpable	Heterozygous	PTPN11	-2.83	–	-0.11	0.27	-2.42	-0.03	–	-2.95	-2.79	13.0 (2.3–11.0)
c.1381G > C(ENST_00000371438)	Male	11.08	Bilateral radial abnormalities	Heterozygous	RUNX2	-2.54	–	–	-0.32	-1.13	–	–	-1.73	-1.53	13.0 (5.6–16.0)
c.1697C > T(ENST_00000264731)	Male	12.83	Nil	Heterozygous	TP63	-4.31	-4.25	-1.72	-4.12	-3.91	-2.9	–	-3	-3.91	5.1 (5.6–16.0)

The characteristics of the participants with pathogenic and likely pathogenic mutations are shown.

Abbreviations: ALS, acid-labile subunit; BMI, body mass index; HC, head circumference; IGF-1, Insulin-like growth factor 1; IGFBP3, Insulin-like growth factor-binding protein 3; SDS, standard deviation score; SNP, single nucleotide polymorphism.

Our approach of selecting those which were present in cases but not controls, were exonic, had a MAF <2%, where carriage of the variant allele fitted the mode of inheritance of the known SS disorder and for missense variants, only including those predicted to be potentially damaging allowed identification of 42 potentially pathogenic variants. After applying the criteria from the American College of Medical Genetics and Genomics and the Association for Molecular Pathology [13] to these 42 variants, 2 were classified as pathogenic, 25 as likely pathogenic, and 6 as VUS. A further 9 were insertions or deletions, where there was insufficient information to allow for classification. Of these, 3 are at locations where there are known variants: rs373470699 in *ACAN*, rs1553765674 in *SF3B4*, and rs755773799 in *SOST*.

### Gene-based Burden Testing: SNP Carriage Frequencies for All Variants

Thirty genes were identified (Table 4) where SNP carriage frequencies across the whole gene were significantly different (corrected *P* ≤ 0.05) between the homozygous/heterozygous variants and wild type. In SS-UA, SNP frequencies were increased in 12 genes and decreased in 18. These included genes that are associated with SS conditions, genes associated with growth pathways, and genes associated with adult height, and the most significant in each of these groups were *FANCA*, *PRKCH*, and *PTCH1*, respectively (all decreased in SS-UA cases).

**Table 4.** SNP carriage frequencies

Gene and Corrected *P*-Values		
SS Disease	Growth Pathway	Adult Height
FANCA (↓) p = 2.54E-17	PRKCH (↓) p = 1.68E-10	PTCH1 (↓) p = 3.47E-07
FANCD2 (↓) p = 1.38E-08	LRP5 (↑) p = 2.34E-05	ATXN3 (↑) p = 2.34E-05
CUL7 (↓) p = 5.61E-05	COL1A1 (↓) p = 7.13E-05	ZBTB38 (↓) p = 4.73E-04
FANCM (↓) p = 7.74-05	SOCS2 (↑) p = 9.82E-0.4	RNF135 (↓) p = 1.97E-03
IGFALS (↓) p = 4.73E-04	CSNK2A2 (↓) p = 6.73E-03	DOT1L (↑) p = 2.97E-03
SOS2 (↑) p = 1.01E-03	PEX2 (↓) p = 2.11E-02	TEAD3 (↑) p = 9.79E-03
STAT5B (↓) p = 1.34E-02	A2M (↓) p = 1.19E-02	SDR16C5 (↑) p = 1.34E-02
MAP2K1 (↑) p = 1.68E-02	RPS6KA1 (↓) p = 1.88E-02	BCAS3 (↓) p = 1.88E-02
CEP63 (↑) p = 1.88E-02	SOCS1 (↑) p = 1.88E-02	EFEMP1 (↓) p = 3.61E-02
HRAS (↑) p = 4.24E-02	BUB1B (↓) p = 3.85E-02	PPARD (↑) p = 4.24E-02

SNP/indel frequency was assessed for carriage of homozygous plus heterozygous variants vs wild type. 30 genes were identified where SNP carriage frequencies were significantly different, with a Bonferroni-corrected *P*-value of ≤0.05. In SSUA SNP frequencies were increased in 12 genes and decreased in 18. These included genes which are associated with short stature conditions, genes associated with growth pathways and genes associated with adult height. The most significant genes in each of these groups were *FANCA*, *PRKCH*, and *PTCH1*.

### Gene-based Burden Testing: SNP Carriage Frequencies When Assessing Rare Variants Alone

When we repeated this analysis for rare variants alone, 19 genes were identified where SNP carriage frequencies were significantly different (corrected *P* ≤ 0.05) between homozygous/heterozygous variants and wild type (Table 5). In SS-UA, SNP frequencies were increased in 3 genes and decreased in 16. The most significant in each of the gene groups were *BUB1B*, *ELK1*, and *PTCH1*, respectively (all decreased in SS-UA).

**Table 5.** Rare variants: SNP carriage frequencies of homozygous plus heterozygous variants vs wild type variants

Gene and Corrected *P*-values		
SS Disease	Growth Pathway	Adult Height
NPR2 (↓) p = 7.22E-04	BUB1B (↓) p = 1.59E-07	PTCH1 (↓) p = 7.22E-04
TRIP11 (↓) p = 1.55E-03	ELK1 (↓) p = 5.18E-03	NHEJ1 (↓) p = 4.85E-03
FANCM (↓) p = 1.18E-02	DGKE (↑) p = 9.01E-03	TEAD3 (↑) p = 7.08E-03
CUL7 (↓) p = 1.60E-02	IGFBPI (↓) p = 4.63E-02	NFKB1 (↓) p = 1.03E-02
FANCA (↓) p = 5.04E-02	PEX2 (↓) p = 4.63E-02	SH2B1 (↑) p = 1.03E-02
		LEPRE1 (↓) p = 2.95E-02
		MC4R (↓) p = 5.04E-02
		BCAS3 (↓) p = 5.04E-02
		DLEU7 (↓) p = 5.04E-02

For those variants with a minor allele frequency of < 2%, SNP/indel frequency was assessed for carriage of homozygous plus heterozygous variants versus wild type. Nineteen genes were identified where SNP carriage frequencies were significantly different, with a Benjamini-Hochberg-corrected *P*-value of ≤ 0.05. In SS-UA, SNP frequencies were increased in 3 genes and decreased in 16. These included genes that are associated with SS conditions, genes associated with growth pathways, and genes associated with adult height. The most significant genes in each of these groups were *NPR2*, *BUB1BI,* and *PTCH1*.

No genes were identified in which the frequency of carriage of an indel significantly differed.

### Single Nucleotide Polymorphisms Associated With Phenotypic Characteristics

**Figure 1.**
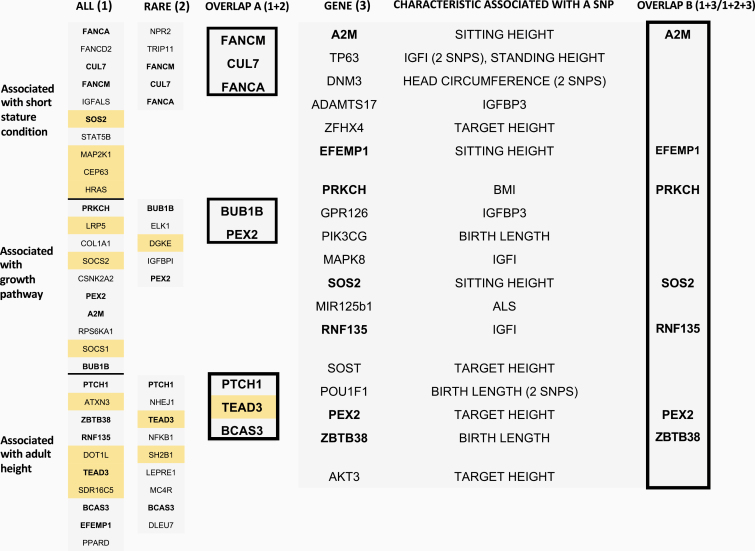
Overlaps between both gene-wide analyses and with genes containing variants associated with growth characteristics/IGF status. Results from both gene-based burden testing approaches are shown here, with overlap A illustrating genes that were identified by both methods. Orange boxes indicate genes where SNP frequency was greater in SS-UA cases and blue boxes represents genes where SNP frequency was lower in SS-UA cases compared with controls. Genes involved in an overlap are shown in bold. On the right, results of the PLINK analyses, linking specific variants to particular growth characteristics/biochemical measures of IGF status are shown. A *P*-value of ≤ 0.001 was considered significant. Overlap B highlights those genes that were identified by this method, as well as by one (all SNPs or rare SNPs alone) or both SNP burden testing approaches.

For each growth characteristic/measure of IGF status, the most significant SNP associations were identified in alpha-2-macroglobulin (*A2M*), tumour protein p63 (*TP63*), and dynamin 3 (*DNM3*), where the presence of the variant allele was associated with shorter sitting height, lower IGF-1, and smaller head circumference, respectively (Table 6 and Fig. 1).

**Table 6.** SNPs associated with growth characteristics/IGF status

Clinical/Biological Characteristic	Gene	Position	Corrected *P*-Value
Sitting height (↓)	A2M	ENSG_00000175899:g.9110456A > C(Chr12, NCBI38)	0.01621
IGF1 (↓)	TP63	ENSG_00000073282:g.189873072G > A(Chr3,NCBI38)	0.04648
HC (↓)	DNM3	ENSG_00000005339:g.172407492C > T(Chr3,NCBI38), rs10752946	0.04971

Three haplotype tagging SNPs were significantly associated with sitting height, IGF-I, and head circumference, which were all decreased. *A2M* encodes alpha-2-macroglobulin, a protease inhibitor and cytokine transporter. *TP63* encodes tumour protein p63, a member of a family of transcription factors. *DNM3* encodes a member of a family of GTP binding proteins that associate with microtubules and are involved in vesicular transport.

One SNP in *A2M* was associated with shorter sitting height (-4.0 SDS in those carrying a variant vs -2.6 SDS in wild type). There was a significant association when no quality control measures were applied (*P* = 7.56 × 10^–6^), when controlling for ethnicity by examining individuals with homogeneous Caucasian ancestry alone (*P* = 1.61 × 10^–5^), and also when the principal component analysis (PCA) was applied to control for ethnicity, age, and sex (*P* = 3.82 × 10^–5^).

An SNP in *TP63* was associated with lower IGF-1 levels before quality control measures (-2.7 vs -1.9, *P* = 2.65 × 10^–5^). This was also significant when analysis was limited to those with homogeneous Caucasian ancestry (*P* = 2.99 × 10^–5^) and following PCA (*P* = 2.49 × 10^-5^).

A variant in *DNM3* was associated with a smaller head circumference (-2.2 vs -1.4, *P* = 6.21 × 10^-5^). This was also found in individuals with homogeneous Caucasian ancestry (*P* = 2.63 × 10^-5^) and when PCA was performed (*P* = 6.21 × 10^-5^).

### Overlaps between Results from SNPs Associated with Phenotypic Characteristics and Both Gene-based Burden Testing Approaches

Figure 1 illustrates genes that were identified by both gene-based burden testing approaches (overlap A), as well as overlaps between genes containing SNPs associated with phenotypic characteristics and one or both gene-based approaches (overlap B).

*PEX2* was the only gene identified by all these methods. An additional 7 genes (*BUB1B, FANCM, CUL7, FANCA, PTCH1, TEAD3, BCAS3*) were identified by both gene-based burden testing approaches (overlap A). A further 6 genes (*A2M, EFEMP1, PRKCH, SOS2, RNF135, ZBTB38*) were identified by gene-based burden testing for all (but not rare) variants, and also contained SNPs associated with phenotypic characteristics.

Of note, following gene-based burden testing for all variants, the 3 most significant genes (*FANCA*, *PRKCH*, and *PTCH1)* in each of the 3 candidate gene selection groups were identified as genes of interest when comparing overlaps, as described above.

## Discussion

In this large, European, multicenter, multiethnic cohort of prepubertal children with SS-UA, in whom clinical assessment and investigations had failed to result in a diagnosis, the search for mutations in genes known to be associated with SS led to a positive yield of 10% (27/263).

We found several previously described mutations, highlighting a need to maintain a degree of suspicion for particular disorders when evaluating a child with SS. In particular, skeletal dysplasias (*ACAN*, *MMP13* mutations) and Noonan syndrome (*PTPN11*) were detected.

Furthermore, by studying overlaps, we identified 14 genes of particular interest in SS-UA. These include genes related to Noonan syndrome (*SOS2*), sitting height (*A2M*), and skeletal disproportion (*BCAS3*), further emphasizing the need to closely evaluate skeletal phenotype when assessing a child with SS.

Of note, *PEX2* was identified by both gene-based burden testing approaches, and an SNP in *PEX2* was significantly associated with target height. This may extend the spectrum of phenotypes associated with this gene.

### Diagnostic Yield of Targeted NGS Panels in Children with SS-UA

Previous studies using targeted NGS panels in SS-UA have resulted in diagnostic yields of 2% to 16.5%. Genes identified included *SHOX, ACAN, NPR2*, and genes known to be involved in the GH–IGF-1 axis [18–21].

Using similar selection criteria to those used in EPIGROW to identify candidate genes, Wang et al [22] performed targeted NGS sequencing in 1077 genes, in 192 children with SS-UA. Four known pathogenic variants were identified where the suggested diagnosis matched the phenotype of the patient, giving a diagnostic yield of 2%. Three individuals had mutations in *PTPN11* indicating Noonan syndrome, and 1 had a *TRPV4* mutation associated with brachyolmia type 3, an autosomal dominant skeletal disorder. A further 64 pathogenic mutations were found that did not match the phenotype. Further work by Wang et al [23] on the cohort of 192 children identified patients with variants in *NPR2*, increasing the diagnostic yield to 13.6% in those with familial SS.

In a study by Hauer et al [24], phenotyping and targeted diagnostic testing of 565 individuals with unexplained SS gave a diagnostic yield of 13.6%. Whole exome sequencing of 200 representative individuals with no determined cause for SS improved the diagnostic yield to 33%. A total of 134 children had ISS, and an additional 66 had syndromic SS.

In a smaller study of 86 individuals, following exome sequencing of 10 genes that are frequently identified in patients with SS-UA (but excluding *SHOX*), Hattori et al [25] identified 4 probably damaging variants in 4 (5%) patients. These were all in *ACAN*. A further 6 variants of unknown pathogenicity were identified in 6 patients (7%). The lower diagnostic yield generated in this study may be a result of the limited candidate gene panel of 10, compared with the 69 known SS genes included in our study.

### Mutations Identified in This Cohort

Two variants in 1 gene, *ACAN*, were classed as pathogenic. An additional 25 variants in 15 genes were classed as likely pathogenic. These were in *FANCB*, *GDF5*, *GH1*, *GNAS*, *HRAS*, *IGF1R*, *IKBKG*, *LHX4*, *MMP13*, *NOG*, *NPR2*, *OBSL1*, *PTPN11*, *RUNX2*, and *TP63*. This highlights particular conditions to consider when assessing a patient with SS-UA and extends the phenotype of known diseases associated with these genes (Tables 2 and 3).

We found 2 heterozygous nonsense mutations in *ACAN. ACAN* encodes the proteoglycan, aggrecan, which is present in the extracellular matrix of the growth plate. Patients may present with SS and advanced bone age, resulting in a mild skeletal dysplasia [26, 27]. More recently, there has been conflicting evidence that patients with *ACAN* variants may display SS and age-appropriate or even delayed bone age. Additionally, since aggrecan is present in cartilaginous tissue [28], cartilaginous pathology (early-onset arthritis and intervertebral disc disease) has been observed. An analysis of 428 families with SS-UA revealed that 1.4% had *ACAN* mutations. Within our cohort, only 0.8% (2/263) had *ACAN* mutations. Paucity of data regarding a further 3 *ACAN* indels, 1 of which is at a location where a known variant is present (rs373470699), limited our ability to classify these, and this may account for the discrepancy.

### Genes of Interest: Overlaps between Genes Identified by Gene-based Burden Testing for All Variants and for Rare Variants

As indicated by Fig. 1 (overlap A), 8 genes were identified by both gene-wide approaches. These were *BCAS3*, *BUB1B*, *CUL7*, *FANCA*, *FANCM*, *PEX2, PTCH1*, and *TEAD3* (see supplemental material) [12].

### Single Nucleotide Polymorphisms Associated with Phenotypic Characteristics

The finding of an association between *A2M* and sitting height, as well as a difference in SNP carriage frequency when all (not only rare) genes were analyzed, may suggest that this gene plays a role in the aetiology of skeletal disproportion. Whilst our cohort was selected to exclude these cases, it is possible that children with subtle undiagnosed skeletal dysplasias were enrolled. This may highlight the importance of routine sitting height measurement in children presenting with SS.

In individuals with homogeneous Caucasian ancestry, a polymorphism in *DNM3* was associated with a smaller head circumference. Although this has not been previously described, *DNM3* has been implicated in 1q24 deletion syndrome, in which microcephaly is a feature.

An SNP in *TP63*, a cell cycle regulator, was associated with the IGF-1 level. This is a novel finding.

### Further Genes of Interest: Overlaps between Genes Identified from Phenotype Associations and Both Gene-based Burden Testing Approaches

As shown in Fig. 1 (overlap B), an additional 6 genes were identified by the gene-based burden testing approach for all variants that also contained SNPs associated with phenotypic characteristics. These were *A2M*, *EFEMP1*, *PRKCH*, *RNF135*, *SOS2*, and *ZBTB38* (see Supplemental Table [12]).

### Primary Gene of Interest: PEX2

As shown in Fig. 1, *PEX2* was the only gene that was identified by both gene-wide approaches and that was also significantly associated with a growth characteristic—target height. In both gene-wide analyses, the frequency of SNPs in *PEX2* was lower in cases compared with controls.

*PEX2* encodes peroxisome biogenesis factor 2. This has been implicated [29] as the E3 ubiquitin ligase that signals pexopaghy, an autophagic process that regulates peroxisome numbers. Sargent et al [29] found an upregulation of *PEX2* expression during treatment with rapamycin and suggested that the mammalian target of rapamycin complex 1 (mTORC1, which is inhibited by rapamycin) regulates pexophagy by controlling *PEX2* expression levels.

Following prolonged exposure, rapamycin may also inhibit mTOR complex 2 (mTORC2) in some cell lines [30]. mTORC2 acts as a tyrosine protein kinase, phosphorylating and thereby activating IGF-1R and insulin receptor substrate-1. In doing so, it plays a key role in cell proliferation and growth [31].

We found that in using both gene-based burden testing approaches, the frequency of SNPs in *PEX2* was lower in cases than in controls. We postulate that the involvement of the mTOR pathway links the observation of a lower frequency of SNPs in SS-UA subjects to their SS. We also discovered an SNP in *PEX2* was associated with target height. Previous studies have associated *PEX2* with the timing of the onset of puberty [32]. Moreover, a GWAS meta-analysis of 18 737 European individuals [33] found that a genetic locus in *PEX2* was associated with pubertal height and growth rates. Since pubertal timing tends to be familial, this could potentially account for the association with target height. In contrast, a study of 600 individuals that selected particular SNPs associated with age at menarche in females, including *PEX2*, did not find an association with change in height [34].

In this cohort, no pathogenic or likely pathogenic variants were found in *PEX2*. Mutations in *PEX2* are implicated in metabolic disease, which extends from Zellweger syndrome (most severe) to neonatal adrenoleukodystrophy (intermediate) and infantile Refsum disease (currently considered the mildest) [35].

Our previous report of *NDUFB3* mutations manifesting as SS, with a relatively benign clinical course compared with the life-threatening lactic acidosis, which can result, provides a similar example of mild metabolic phenotype presenting with SS-UA [36].

### Strengths

EPIGROW was the first European epidemiogenetic study that investigated the clinical and biological characteristics of children with SS-UA [10].

The data generated from the EPIGROW study resulted in a rich dataset, which we have analyzed using new approaches. Firstly, we identified previously known and novel mutations. Whilst this panel of 69 growth-related genes may be limited compared with other studies [22], the diagnostic yield that we generated was similar. We went on to identify a number of participants with SNPs suggestive of specific SS conditions, where standard clinical assessment and investigations had not led to a diagnosis. In doing so, we also highlighted particular conditions that should be considered when clinically assessing a child with SS-UA. Furthermore, by identifying mutations in children exhibiting SS without classical signs (eg, *ACAN*), we have extended the spectrum of phenotypes associated with these conditions.

Secondly, we used 2 gene-based burden testing approaches and identified SNPs associated with phenotypic characteristics, which allowed us to identify particular genes of interest that may contribute to SS in children with SS-UA.

Gene-based burden testing is a strategy that has been successfully validated in both polygenic traits [37–39] and monogenic disorders [40]. As an example, Guo et al [40] compared the frequency of qualifying variants between cases and controls, where a “qualifying variant” was defined following a series of filters (prediction of functional consequence and minor allele frequency suggestive of a rare variant). We initially assessed all SNPs in each of the 232 candidate genes, but our second gene-based approach included only rare variants (MAF < 2%).

Using PLINK software to assess for associations between SNPs and phenotypic characteristics, we were able to apply a number of quality control measures prior to performing the analysis. The associations observed passed a stringent experiment-wide correction for significance, increasing the likelihood that the genes we identified have a genuine relationship with particular growth characteristics in this cohort of children with SS-UA.

### Limitations

A number of limitations relate to these analyses being performed on a dataset that was generated from the original EPIGROW study in 2008. Firstly, while it may not be appropriate to apply today’s standards to NGS data from 12 years earlier, the analysis, using a candidate gene approach, would have benefited from a higher vertical coverage of genomic regions. Secondly, in comparison to GWAS, our sample size of 263 cases was relatively small. To mitigate this, our subjects were carefully selected, we used a knowledge-based selection of 232 candidate genes from functional-, pathway-, and height-related studies [10], and our sample size was similar to other studies that have established a diagnostic yield [22, 24] in SS-UA. Thirdly, our subjects were enrolled from across Europe, and although they were ethnically matched to anonymous controls, rare variant frequencies may have differed across the countries and to the controls.

To identify associations, we used PLINK, a genome-wide association analysis toolset. Whilst PLINK has value in handling large datasets, analysis of indels was more challenging, particularly because our data relating to the specific indels were limited. More detail may have also allowed us to classify the indels as pathogenic or likely pathogenic.

Paucity of phenotypic data relating to specific conditions also limited our ability to relate genotype and phenotype data. For example, phenotypic data regarding radial and/or blood count abnormalities are not available for a participant with a homozygous FANCB mutation, nor are parental genotypes. Also, whilst the control group were ethnically matched, healthy adult controls, there are no data available to characterize this group further.

## Conclusions

This gene panel led to a diagnostic yield of 10% (27/263) in a cohort of prepubertal children with SS-UA. Therefore, the use of such panels may improve diagnosis in SS-UA and extend the phenotype of known diseases.

In this large European cohort of children with SS-UA, we have identified 14 genes that are of particular relevance and that could be candidates for future, more detailed genetic investigation.

In particular, RASopathies and skeletal disproportion syndromes should be considered. Therefore, sitting height measurement is a valuable clinical tool in the assessment of a child presenting with SS.

## Additional Information

***Disclosure Summary:*** Professor Clayton was the chief investigator for the original EPIGROW study, which was funded by Ipsen. A draft of the manuscript was provided to Ipsen for courtesy review.

***Data Availability:*** The datasets generated during and/or analyzed during the current study are not publicly available but are available from the corresponding author on reasonable request.
